# Economical Assessment of Working Memory and Response Inhibition in ADHD Using a Combined *n*-back/Nogo Paradigm: An ERP Study

**DOI:** 10.3389/fnhum.2020.00322

**Published:** 2020-08-06

**Authors:** Carolin Breitling-Ziegler, Jana Tegelbeckers, Hans-Henning Flechtner, Kerstin Krauel

**Affiliations:** ^1^Department of Child and Adolescent Psychiatry and Psychotherapy, Otto von Guericke University Magdeburg, Magdeburg, Germany; ^2^Department of Neurology, Feinberg School of Medicine, Northwestern University, Chicago, IL, United States; ^3^Center for Behavioral Brain Sciences, Otto von Guericke University Magdeburg, Magdeburg, Germany

**Keywords:** attention-deficit/hyperactivity disorder (ADHD), working memory, response inhibition, *n*-back task, go/nogo task, economical assessment, dual-task

## Abstract

The development of cognitive interventions in attention-deficit/hyperactivity disorder (ADHD) often requires the assessment of multiple cognitive functions. However, experimental settings consisting of various tasks are particularly strenuous for patients and can thus result in poor data quality. For the economical assessment of working memory and response inhibition, this study aims to validate a combined *n*-back/nogo paradigm by comparing it to single task versions and to demonstrate its applicability for ADHD research. Twenty-five healthy individuals and 34 ADHD patients between 9 and 16 years participated in this event-related potential (ERP) study. Healthy controls underwent single task versions of a 2-back working memory task and a go/nogo response inhibition task as well as the introduced combined 2-back/nogo task. This combined task demonstrated a comparable ERP structure for working memory and response inhibition aspects as single task versions. Behaviorally, higher working memory performance during the combined paradigm indicated lower task difficulty, while high correlations between combined and single task versions still indicated valid working memory measures. For response inhibition performance, different task versions resulted in similar outcomes. The application of the combined *n*-back/nogo paradigm in ADHD patients revealed the expected working memory and response inhibition deficits, increased omission errors, reaction times, and standard deviation of reaction time, as well as diminished *n*-back P3 and nogo P3 amplitudes. We conclude that the combined *n*-back/nogo task is an effective paradigm for the economical assessment of working memory and response inhibition deficits in ADHD on a behavioral and neurophysiological level.

## Introduction

Attention-deficit/hyperactivity disorder (ADHD) is one of the most frequent disorders in child and adolescent psychiatry, with a worldwide prevalence of about 3.4% (Polanczyk et al., 2015). Additional to the core symptoms of inattention, hyperactivity, and impulsivity defined in (DSM-5; American Psychiatric Association, 2013), 62% of ADHD patients are affected by significant impairments of working memory and 27% suffer from inhibitory dysfunctions (Kofler et al., 2019). These deficits are associated with long-term consequences as academic underachievements, social problems and even addiction (Groman et al., 2009; Rinsky and Hinshaw, 2011; Simone et al., 2018). As those impairments mostly persist into adulthood (Barkley and Murphy, 2011), we emphasize that working memory and response inhibition can be essential indicators for the success of therapeutic interventions.

Therapeutic approaches that aim for the improvement of cognitive functions in ADHD include cognitive training (Johnstone et al., 2010), neurofeedback (Baumeister et al., 2018), and non-invasive brain stimulation (Salehinejad et al., 2019; Breitling et al., 2020). When evaluating the effectiveness of such interventions, the assessment of multiple cognitive functions, as working memory and response inhibition, can be necessary not only to demonstrate therapeutic success but also to detect transfer effects into other domains or to control for cognitive side effects. However, experimental settings consisting of various tasks are particularly challenging for ADHD patients who are unable to stay concentrated and calm over longer time periods (Dekkers et al., 2017). In imaging studies, this is a highly relevant obstacle because data quality is suffering when patients start to fidget or move. Thus, researchers often refrain from the assessment of multiple cognitive functions or accept poor data quality. With this motivation, we applied the approach of merging different tasks (Ruchsow et al., 2008; Lee et al., 2010; Scharinger et al., 2015; Alderson et al., 2017) to a combined working memory and a response inhibition paradigm.

One of the most popular paradigms in working memory research is the *n*-back task. During this task, participants decide for a series of stimuli if the current stimulus is identical to the one presented a specified number *n* of trials earlier. With increasing *n* and therefore increasing working memory load, the task becomes more demanding. There is strong evidence that ADHD patients show impaired performance as well as higher reaction time and standard deviations of reaction time during *n*-back tasks (Keage et al., 2008; Myatchin et al., 2012) with largest effect sizes of impairments during the 2-back variant (Kobel et al., 2009). Response inhibition is commonly investigated using the go/nogo task. In this task, participants are instructed to react fast on pre-defined go stimuli and to withhold their reaction for nogo stimuli. Performance deficits as well as increased reaction time and standard deviations of reaction time in ADHD patients were found in this task (Fallgatter et al., 2004; Wiersema et al., 2006; Fisher et al., 2011). As neuropsychological deficits in ADHD were effectively demonstrated using 2-back and go/nogo paradigms, we combined both into the *n*-back/nogo task.

The investigation of this combined *n*-back/nogo paradigm was realized by assessing behavioral parameters and electroencephalograms (EEG) during the application of single and combined task versions. EEG analysis is optimally suited to investigate neural mechanisms underlying behavioral data. For that purpose, we focused on the following event-related potential (ERP) components as they represent the executive processes that show pathological changes in ADHD (Barry et al., 2003; Banaschewski and Brandeis, 2007).

The working memory *n*-back task evokes a P3 component that peaks at parietal sites and that is larger during *n*-back targets compared to non-targets (Watter et al., 2001). This component represents the amount of resources allocated to the working memory process (Keage et al., 2008). The P3 amplitude decreases with increasing *n* because higher working memory load results in the distribution of cognitive resources to meet task requirements (Watter et al., 2001). Furthermore, larger P3 amplitudes were found in individuals with better working memory (Dong et al., 2015). In accordance with that, ADHD patients show diminished *n*-back P3 amplitudes (Stroux et al., 2016) indicating reduced resource allocation to working memory processing (Keage et al., 2008).

The go/nogo response inhibition task elicits a frontal N2 and a fronto-central P3 component, which are both larger during nogo than during go trials (Smith et al., 2008). The nogo N2 component reflects conflict monitoring (Donkers and van Boxtel, 2004), which is considered a non-motoric subprocess of inhibition (Smith et al., 2008). Larger N2 amplitudes and lower latencies of this component are associated with better inhibitory performance (Barry et al., 2003). In line with that, reduced peaks of the nogo N2 were found in ADHD patients, resulting from atypical inhibitory processes in frontal areas (Barry et al., 2003). However, results regarding N2 latencies in ADHD patients are inconclusive, as different studies found either reduced (Smith et al., 2004; Johnstone et al., 2009) or increased latencies (Barry et al., 2003; Fallgatter et al., 2004; Fisher et al., 2011), or found no ADHD related changes (Barry et al., 2003; Fallgatter et al., 2004). The nogo P3 component reflects response inhibition (Donkers and van Boxtel, 2004) and is thus a marker for the success of motoric inhibition (Smith et al., 2008). Accordingly, in healthy adults with high impulsivity reduced nogo P3 amplitudes have been demonstrated (Ruchsow et al., 2008). Reduced amplitudes of the nogo P3 component are one of the most robust ERP findings in ADHD (Kaiser et al., 2020) and have been associated with response inhibition impairments in ADHD patients (Johnstone et al., 2013).

The present study aimed to validate the introduced, combined *n*-back/nogo paradigm and to demonstrate its applicability as a measurement for cognitive impairments in ADHD patients in a two-step approach. First, healthy children and adolescents performed the combined *n*-back/nogo task as well as parallel single task versions of *n*-back and go/nogo. We hypothesized that behavioral measures and ERP characteristics would be comparable between task versions. Second, ADHD patients underwent the combined *n*-back/nogo task to demonstrate similar behavioral and neurophysiological deficits as expected from established single task versions. We predicted that patients would show impaired working memory and response inhibition performance as well as reduced amplitudes of *n*-back P3, nogo N2, and nogo P3 components.

## Materials and Methods

### Participants

Fifty-nine participants aged between nine and 16 years were recruited via the Department of Child and Adolescent Psychiatry and through advertisements in a local newspaper. Thirty-four were diagnosed with ADHD according to DSM-5 criteria (21 combined presentation, 12 predominantly inattentive presentation, one predominantly hyperactive-impulsive presentation). Patients with comorbid psychiatric or neurologic diagnoses were excluded from the study. This also applied to dissocial disorders, because differential ERP patterns were demonstrated between patients with ADHD only and patients with comorbid diagnoses as oppositional defiant disorder or conduct disorder (Banaschewski et al., 2003). Eleven patients currently took ADHD medication but they refrained at least 24 h before the experiment. For assignment to the healthy control group, participants had to be free of psychiatric and neurologic disorders (*n* = 25). Trained psychologists diagnosed participants on the basis of clinical interviews, which were conducted with all participants and their parents using the German Adaptation (Delmo et al., 2000) of the Schedule for Affective Disorders and Schizophrenia for School-Age Children – Present and Lifetime Version (K-SADS-PL, Kaufmann et al., 1997). As supportive diagnostic information, concentration performance was determined (d2, Brickenkamp, 2002; d2-R, Brickenkamp et al., 2010) and behavioral problems were assessed in all participants as parent rating (Child Behavior Checklist, Achenbach, 1991a), and additionally in children of 11 years and older as self-rating (Youth Self Report, Achenbach, 1991b). IQ values below 80 served as an exclusion criteria, assessed with the CFT 20-R (Weiss, 2008). Last, the Edinburgh Handedness Inventory (Oldfield, 1971) was applied. Table 1 summarizes sample characteristics and shows that ADHD and control group did not significantly differ in the proportion of females, age, intelligence or handedness (all *p* ≥ 0.085) but in subjective and objective assessment of attentional impairments (all *p* < 0.001).

**TABLE 1:** Sample characteristics of ADHD and control group, mean ± standard deviation, effect size Cohens *d*, *t*, and *p*-values are given.

	ADHD	Controls	*t*	*p*	*d*
*n*	34	25	–	–	–
Females in %	20.6	20.0	–	0.956	–
Age in years	13.15 ± 1.89	13.00 ± 2.02	–0.28	0.775	0.08
Ethnicity in %			–	0.466	–
Caucasian	91.2	96.0	–	-	–
Biracial	8.8	4.0	-	-	-
**Diagnoses:**					
ADHD combined	21	–	–	–	–
ADHD inattentive	12	–	–	–	–
ADHD hyperactive	1	–	–	–	–
**Current medication:**					
Methylphenidate	10	–	–	–	–
Dexamphetamine	1	–	–	–	–
Attention problems, parent rating (CBCL; *T*)	67.6 ± 8.2	54.4 ± 7.1	−6.45	<0.001	1.72
Attention problems, self rating (YSR; *T*)	60.9 ± 7.4	52.8 ± 4.2	−4.79	<0.001	1.35
Attentional performance (d2; *T*)	50.4 ± 8.8	59.7 ± 11.5	3.41	0.001	0.91
IQ	102.3 ± 14.4	108.6 ± 12.6	1.75	0.085	0.47
Left-handed in %	2.9	8.0	–	0.382	–

The study followed the ethical standards of the Declaration of Helsinki and was approved by the local ethics committee of the Otto-von-Guericke University Magdeburg. All caregivers and participants gave their written informed consent and assent, respectively. Participants were reimbursed with a voucher of 15–20€ for a local shopping center.

### Tasks and Procedure

**FIGURE 1:**
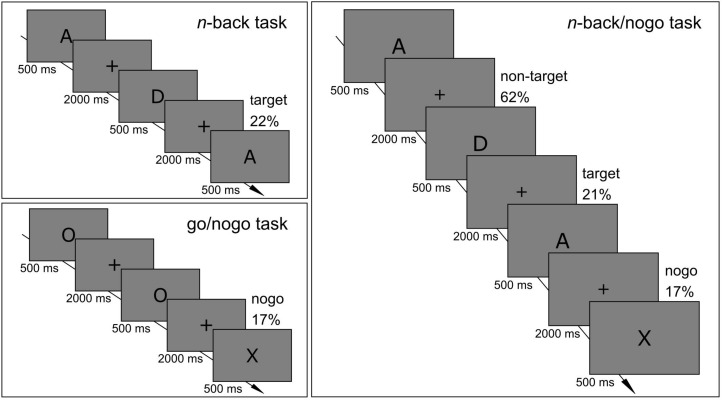
Schematic illustrations of **(A)** single *n*-back task, **(B)** single go/nogo task, and **(C)** combined *n*-back/nogo task.

The healthy control group performed single *n*-back and go/nogo task versions as well as the combined *n*-back/nogo task within one session. The order in which cognitive tasks were applied was pseudo-randomized between participants, with balanced frequency for each possible sequence. ADHD patients underwent only the combined *n*-back/nogo task. Task illustrations are provided in Figure 1. Participants were instructed to react as accurately and as fast as possible. Tasks were presented on a flat screen that had a diagonal of 61 cm using Presentation^®^ (version 18.0)^1^. Stimuli had a visual angle of 0.86° (height) and were presented in black on a gray background (RGB value 128). In all tasks, stimulus duration was 500 ms and participants had 2000 ms to give their response. The interstimulus interval was 2500 ms. The paradigms that were used in this study can be found under https://doi.org/10.6084/m9.figshare.c.4933326.

#### Single *n*-back Task

A series of capital letters was presented (A, D, E, H, I, N, R, S, T, U) and participants decided if the current stimulus was identical to the stimulus two trials earlier (*n* = 2). These target trials had a proportion of 22%. If stimuli were identical, participants pressed a button with their right hand, and if stimuli were not identical, they pressed a button with their left hand. The key assignment was the same for all participants. The task consisted of three runs that had a duration of 3.8 min each (90 trials) and that were separated by pauses of at least 30 s. The task started with a training run of 2.5 min (40 trials) with feedback indicating right or wrong reactions. After this training, the investigator decided if the participant understood the task correctly or if the training must be repeated.

#### Single Go/Nogo Task

Participants were instructed to press a button for go trials presenting the letter O and to withhold their response for nogo trials presenting the letter X (17%). The task consisted of three runs with a duration of 4.6 min each (110 trials). It started with a short training run of 40 s (10 trials) with feedback of right or wrong reactions, which could be repeated if required.

#### Combined *n*-back/Nogo Task

Again, letters were presented sequentially and participants decided if the present and the 2-back stimulus were identical (21%). Additionally, participants were instructed to withhold their response when the letter X appeared. Those nogo trials had a proportion of 17%. The task was composed of a random order of *n*-back sequences containing one to eleven trials (*M* = 5.8, *SD* = 3.0), with the last trial of each sequence always being a nogo trial. Participants were informed that there was never an *n*-back target trial directly after a nogo trial. The task was split into three runs with a duration of 4.6 min each (110 trials), providing an equal quantity of *n*-back target trials to the single *n*-back task. It started with a training run of 2 min (30 trials) with feedback of right or wrong reactions, which could be repeated if required.

Data from ADHD patients were collected in the context of two different studies. During one study, patients performed the combined *n*-back/nogo task in the initial of several sessions for four runs while EEG was recorded (*n* = 24). The other was an application-oriented study, where the task was applied in one of three sessions for six runs during a placebo non-invasive brain stimulation while EEG was recorded during the last three runs (*n* = 10) (Breitling et al., 2020). Data from both studies indicated comparable results as shown in Supplementary Table 1.

### EEG Recording and Analysis

EEG was recorded with a SynAmps amplifier (Neuroscan, Sterling, VA, United States) from 21 channels. For this purpose, Ag/AgCl-electrodes were placed in an EEG cap (Easycap GmbH, Herrsching, Germany), according to the International 10–20 EEG system (Fp1, Fp2, F7, F3, Fz, F4, F8, FT9, FC5, FC6, FT10, C3, Cz, C4, P7, P3, Pz, P4, P8, O1, O2). The ground electrode was placed at position AFz, and the EEG was referenced to linked mastoids. Via two bipolar channels, EOG was recorded with electrodes placed at the outer canthi of both eyes and at sub- and supra-orbital positions. Impedances were kept below 15 kΩ, and data were recorded with a sampling rate of 500 Hz. A high pass filter of 0.05 Hz, a low pass filter of 70 Hz, and a notch filter of 50 Hz were applied online.

We analyzed data with EEGLAB (Delorme and Makeig, 2004) and ERPLAB (Lopez-Calderon and Luck, 2014) in the MATLAB environment (version R2013a, The MathWorks, Inc., Nattick, MA, United States). EEG data were low pass filtered offline with 30 Hz. Epochs ranging from 200 ms pre to 650 ms post-stimulus were extracted from trials with correct responses, relative to the pre-stimulus baseline. Artifactual epochs were removed in a semi-automated way. First, EEGLAB algorithms detected epochs that contained amplitudes exceeding ±100 μV, abnormal trends exceeding 100 μV, or abnormal spectra. Afterward, a trained investigator verified artifact detection and removed trials containing artifacts. Participants with less than ten remaining epochs were excluded from ERP analysis of the respective condition. Thus, two ADHD patients were excluded from analyses of *n*-back target trials and three from analyses of nogo trials. In the control group, 34.3 (*SD* = 7.7) *n*-back targets and 39.3 (*SD* = 6.7) nogo trials were analyzed on average in each participant for single task versions. A mean number of 48.1 (*SD* = 8.5) *n*-back targets and 42.0 (*SD* = 6.9) nogo trials of the combined *n*-back/nogo task remained in the analysis of controls. In ADHD patients, a mean of 38.4 (*SD* = 19.8) *n*-back targets and of 41.3 (*SD* = 14.7) nogo trials were analyzed on average.

ERP peak amplitudes and latencies were determined automatically with ERPLAB measurement tools. Latency ranges for ERP measurements were chosen by reference to grand average waveforms. The time window for the *n*-back P3 analysis was 275–500 ms in both, single *n*-back and combined *n*-back/nogo tasks. The nogo N2 component was analyzed at 225–350 ms in the single go/nogo task and at 250–500 ms in the combined *n*-back/nogo task. For P3 analysis in the single go/nogo task, a time window of 325–600 ms was used for nogo trials and of 225–425 ms for go trials. In the combined *n*-back/nogo task, the nogo P3 was analyzed at 425–625 ms. The *n*-back P3 component has a centro-parietal maximum (Segalowitz et al., 2001) and its analysis was, therefore, restricted to central and parietal electrode positions (C3, Cz, C4, P3, Pz, P4). Analysis of the nogo N2 component focused on frontal and central electrodes (F3, Fz, F4, C3, Cz, C4) as it has a fronto-central distribution (Smith et al., 2008). The nogo P3 was analyzed at frontal, central, and parietal positions (F3, Fz, F4, C3, Cz, C4, P3, Pz, P4).

### Statistics

Statistical analyses were conducted with SPSS (version 24, IBM Corp., Armonk, NY, United States). We compared sample characteristics between the control and ADHD group using independent samples *t*-tests for metric variables, and chi-square tests for dichotomous variables. Reaction times were analyzed from trials with correct responses and with a minimum reaction time of 100 ms. Working memory performance was calculated as the corrected hit rate in percent (hits targets – false positives non-targets) with higher values indicating better performance. Response inhibition performance was the inverse of nogo commission error rate (100% – false positives), thus better performance was indicated by higher values. The analyzed behavioral measures were working memory performance, response inhibition performance, omission errors, reaction time, and standard deviation of reaction times. If Levene’s test for variance equality was significant, Satterthwaite approximations for the degrees of freedom are reported for *t*-tests. ERP components were characterized by peak amplitudes and latencies. These were analyzed using ANOVAs while significant main and interaction effects were further investigated via *post hoc* pairwise comparisons with Bonferroni adjusted alpha levels. We applied Greenhouse–Geisser corrections if assumptions of sphericity were violated. Effect sizes are reported as Cohens *d* for *t*-tests and as η^2^ for ANOVAs.

#### Comparisons Between Single and Combined Task Versions

In the control group, behavioral measures were compared between single tasks and the combined *n*-back/nogo task using paired-samples *t*-tests. Reaction time distributions were characterized and inverse efficiency scores (IES) were determined. The IES is a measure of the speed-accuracy tradeoff, which is defined as the mean reaction time divided by the proportion of correct responses (Liesefeld and Janczyk, 2019). It indicates the reaction time corrected for the amount of errors, thus, smaller values indicate more efficient responses. The convergent validity of the introduced paradigm was investigated by calculating Pearson’s *r* correlation coefficients for working memory and response inhibition performance between single and combined task versions. Further, the correlation between working memory and response inhibition within single tasks and within the combined *n*-back/nogo task were calculated to explore discriminant validity of the introduced paradigm.

For ERP analysis, first, we characterized *n*-back P3 as well as nogo N2 and P3 components within single task versions in healthy controls. For this purpose, repeated measures ANOVAs with the factors Stimulus (go vs. nogo; *n*-back target vs. non-target), Region (*n*-back P3: central vs. parietal; nogo N2: frontal vs. central; nogo P3: frontal vs. central vs. parietal) and Hemisphere (left vs. midline vs. right) were conducted for ERP characteristics. Data for the different levels of Region and Hemisphere were averaged across the following electrodes: frontal – F3, Fz, F4; central – C3, Cz, C4; parietal – P3, Pz, P4; left – F3, C3, P3; midline – Fz, Cz, Pz; right – F4, C4, P4. Second, we tested if components differed between each single task version and the combined *n*-back/nogo task, using repeated measures ANOVAs with the factors Task (single vs. combined), Region, and Hemisphere. Only main and interaction effects including the factor Task were reported for this analysis.

#### Comparisons Between Control and ADHD Group

Behavioral measures during the combined *n*-back/nogo task were compared between the control and ADHD group using independent-samples *t*-tests. Further, the Pearson’s *r* correlation coefficient was calculated between working memory and response inhibition performance within the combined *n*-back/nogo task. ERP components were analyzed in mixed ANOVAs including the between-subjects factor Group (controls vs. ADHD) and within-subjects factors Region and Hemisphere. For this analysis, only main and interaction effects including the factor Group were reported.

#### Power Analysis

*Post hoc* power analyses were conducted using the software G^∗^Power version 3.1 (Faul et al., 2009) to compute the sensitivity of the present study. Given an alpha of 0.05 and a power of 0.80 the present study design allowed for the detection of medium sized effects. Minimal detectable effects for all comparisons are presented in Table 2.

**TABLE 2:** Minimal detectable effects (MDE) for the present study given an α = 0.05 and a power = 0.80, effect sizes are given as Cohens *d* for *t*-tests and as η^2^ for ANOVAs.

Comparison	MDE
***Behavioral data***	
Single vs. combined task	*d* = 0.58
Controls vs. ADHD	*d* = 0.75
***ERP data***	
Single vs. combined task	η^2^ = 0.078
Controls vs. ADHD:	
Between subject	η^2^ = 0.098
Within subject	η^2^ = 0.035
Interaction	η^2^ = 0.035

## Results

### Comparisons Between Single and Combined Task Versions

#### Behavioral Data

Comparisons between task versions indicated better performance for the combined *n*-back/nogo task compared to single task versions. So, better working memory performance was found during the combined *n*-back/nogo task than during the single *n*-back task [combined *n*-back/nogo: 73.83%, single *n*-back: 55.70%, *t*(24) = 6.91, *p* < 0.001, *d* = 1.11]. Response inhibition performance was not significantly different between task versions [combined *n*-back/nogo: 87.44%, single go/nogo: 83.02%, *t*(24) = 1.76, *p* = 0.091, *d* = 0.44] Further, we found a borderline significant trend toward a reduced number of omission errors in the combined *n*-back/nogo task compared to the single *n*-back task [*t*(24) = −2.04, *p* = 0.053, *d* = 0.43]. Reaction times and standard deviations of reaction times for the combined *n*-back/nogo task were significantly higher than for go stimuli of the single go/nogo task (all *p* < 0.001, *d* ≥ 1.84) and were rather comparable to the single *n*-back task (all *p* ≥ 0.338, *d* ≤ 0.18). Table 3 summarizes behavioral results.

**TABLE 3:** Comparisons within the healthy control group between single task versions of *n*-back and go/nogo against the combined *n*-back/nogo task, mean ± standard deviation *t* and *p*-values as well as effect sizes Cohens *d* are given.

	Single task	*n*-back/nogo	*t*(24)	*p*	*d*
***Working memory***					
Performance%	55.7017.29	73.8315.39	6.91	<0.001	1.11
Omission errors in%	2.002.76	1.071.25	–2.04	0.053	0.43
Reaction time in ms	705189	722194	0.59	0.561	0.09
SD of reaction time in ms	26684	25272	–0.98	0.338	0.18
IES in ms	889308	826263	–1.43	0.165	0.22
***Response inhibition***					
Performance in %	83.0211.59	87.448.35	1.76	0.091	0.44
Omission errors in %	1.102.59	1.071.25	–0.07	0.948	0.02
Reaction time in ms	42188	722194	10.30	<0.001	2.00
SD of reaction time in ms	12665	25272	8.77	<0.001	1.84
IES in ms	48082	820176	11.45	<0.001	2.48

**FIGURE 2:**
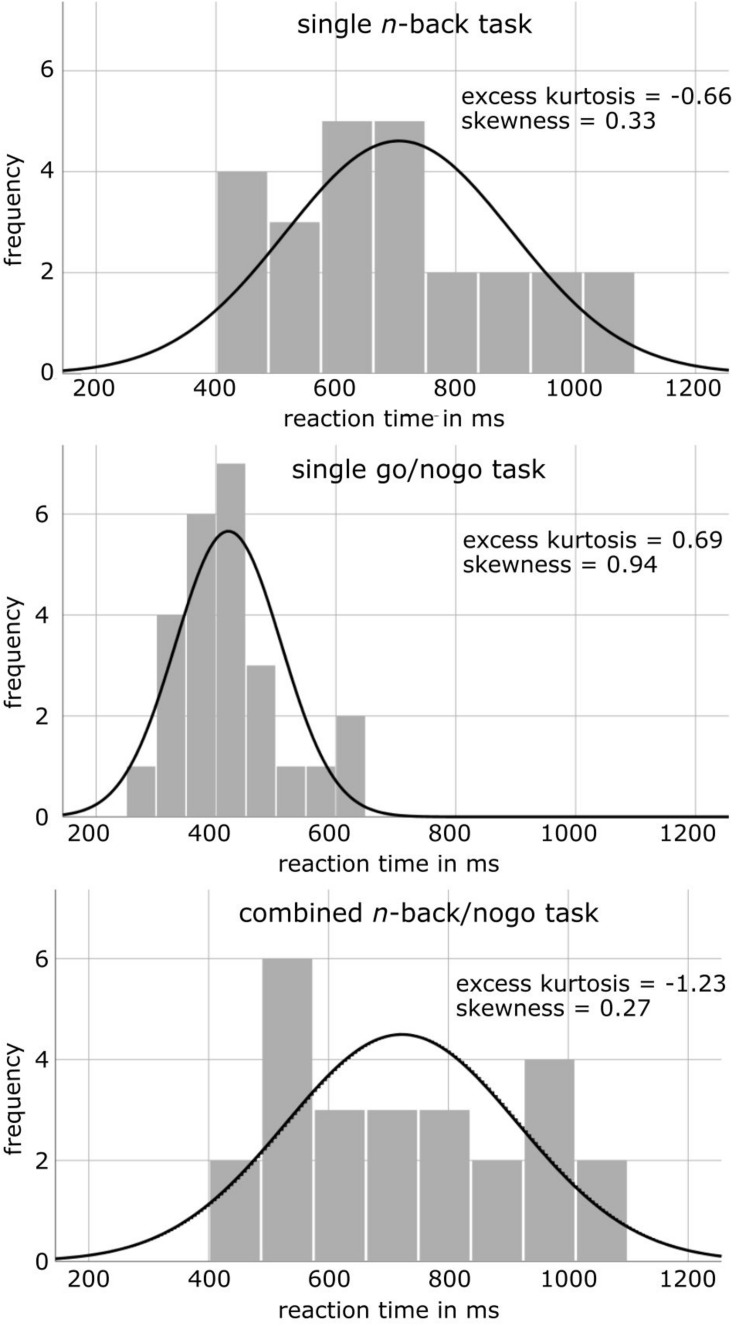
Histograms of reaction time distributions in the healthy control group for different cognitive tasks, together with the illustration of a normal distribution.

As reaction time measures differed significantly between single go/nogo and combined *n*-back/nogo task versions, reaction time distributions were characterized further (Figure 2). While the excess kurtosis of the reaction time distribution was at 0.69 for the single go/nogo task, the distribution flattened in the combined *n*-back/nogo task (excess kurtosis = −1.23) and was thus, more similar to the reaction time distribution of the single *n*-back task (excess kurtosis = −0.66). Further, while the reaction time distribution of the single go/nogo task was moderately skewed left (skewness = 0.94), reaction time distributions were approximately symmetric for the single *n*-back task (skewness = 0.33) and for the combined *n*-back/nogo task (skewness = 0.27). Moreover, regarding the IES we found no significant task difference for working memory performance [single *n*-back: 889 ms, combined *n*-back/nogo: 826 ms, *t*(24) = −1.43, *p* = 0.165, *d* = 0.22], but for response inhibition the IES was higher in the combined *n*-back/nogo task (820 ms) compared to the single go/nogo task (480 ms), [*t*(24) = 11.45, *p* < 0.001, *d* = 2.48].

**FIGURE 3:**
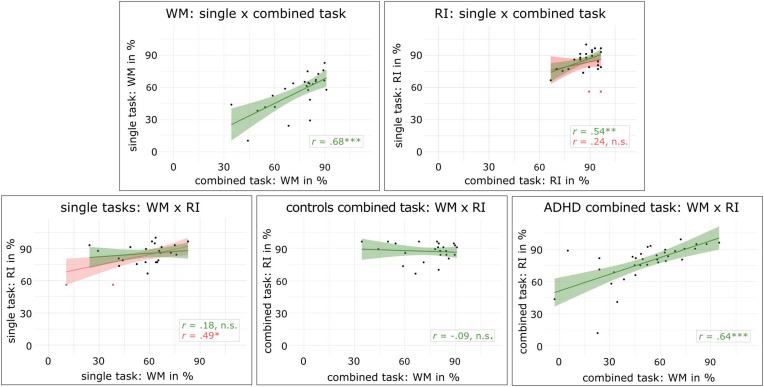
Scatterplots and regression lines with 95% confidence intervals are given for correlations of working memory performance (WM) and response inhibition performance (RI) in different task conditions, red lines represent the results including the two outliers that are marked as red dots, ^∗^*p* < 0.05, ^∗∗^*p* < 0.01, ^∗∗∗^*p* < 0.001.

Pearson’s *r* correlation coefficients of behavioral measures between task versions revealed that working memory performance was highly correlated between the combined *n*-back/nogo task and the single *n*-back task (*r* = 0.68, *p* < 0.001). Further, the number of omission errors in the combined *n*-back/nogo task was correlated to omission errors in the single *n*-back task (*r* = 0.58, *p* = 0.003) as well as in the single go/nogo task (*r* = 0.50, *p* = 0.011). Reaction times in the combined *n*-back/nogo task were correlated to reaction times in both single task versions (*n*-back: *r* = 0.72, *p* < 0.001, go/nogo: *r* = 0.70, *p* < 0.001) and the same applied for standard deviations of reaction times (*n*-back: *r* = 0.59, *p* = 0.002, go/nogo: *r* = 0.45, *p* = 0.023). For response inhibition performance, we found no significant correlation between task versions (*r* = 0.24, *p* = 0.250). However, data inspection revealed two outliers for this correlation (compare Figure 3). When the outliers were removed in an exploratory analysis, a significant correlation of *r* = 0.54, *p* = 0.007 was revealed. Unexpectedly, when exploring measures of discriminant validity, a significant correlation between working memory performance in the single *n*-back task and response inhibition performance in the single go/nogo task was found (*r* = 0.49, *p* = 0.014). However, the same participants as above were identified as outliers and excluded for an exploratory analysis, resulting in no significant correlation (*r* = 0.18, *p* = 0.403). In the combined *n*-back/nogo task, working memory and response inhibition performance were not significantly correlated with each other (*r* = −0.09, *p* = 0.661).

#### ERP Data

##### n-back P3

The comparison of P3 amplitudes between *n*-back targets and non-targets during the single *n*-back task revealed main effects of Stimulus [*F*(1,24) = 45.54, *p* < 0.001, η^2^ = 0.655], Region [*F*(1,24) = 79.25, *p* < 0.001, η^2^ = 0.768], Hemisphere [*F*(1.4,34.0) = 6.72, *p* = 0.008, η^2^ = 0.219], and an interaction between Stimulus and Hemisphere [*F*(2,48) = 3.33, *p* = 0.044, η^2^ = 0.122]. This analysis indicated larger P3 components in response to target than to non-target stimuli and at parietal than at central electrode positions. *Post hoc* tests showed larger amplitudes at midline compared to left electrode sites for target and non-target stimuli (all *p* ≤ 0.007) while only for non-targets amplitudes were larger at right compared to left sites (*p* = 0.011). P3 amplitudes did not significantly differ between single *n*-back and combined *n*-back/nogo tasks [*F*(1,24) = 0.83, *p* = 0.372, η^2^ = 0.033].

Latency comparisons of *n*-back P3 between target and non-target trials in the single task version resulted in an interaction effect between Stimulus and Region [*F*(1,24) = 10.35, *p* = 0.004, η^2^ = 0.301], indicating higher latencies at central than parietal positions during non-target trials (*p* = 0.007). A further interaction between Stimulus and Hemisphere was found [*F*(2,48) = 5.41, *p* = 0.008, η^2^ = 0.184], but *post hoc* tests did not reach significance. Subsequent analyses showed no latency differences between single and combined task versions [*F*(1,24) < 0.01, *p* = 0.964, η^2^ < 0.001].

##### Nogo N2

The comparison of N2 amplitudes between go and nogo stimuli of the single go/nogo task, showed main effects of Stimulus [*F*(1,24) = 7.59, *p* = 0.011, η^2^ = 0.240] and Region [*F*(1,24) = 33.33, *p* < 0.001, η^2^ = 0.581], as well as an interaction between Region and Hemisphere [*F*(2,48) = 7.02, *p* = 0.002, η^2^ = 0.226]. Hence, N2 peaks were more negative for nogo than for go stimuli and at frontal than at central electrode positions. Focusing on frontal sites, amplitudes were more negative at the midline compared to the right electrode (*p* < 0.001) whereas there was no difference between central sites (all *p* = 1.00). Task version did not affect N2 amplitudes [*F*(1,24) = 0.52, *p* = 0.479, η^2^ = 0.021].

During the single go/nogo task, N2 latencies were higher for nogo than for go stimuli [*F*(1,24) = 6.13, *p* = 0.021, η^2^ = 0.204], at frontal than at central electrode sites [*F*(1,24) = 4.71, *p* = 0.040, η^2^ = 0.164], and they were highest at right sites [*F*(2,48) = 4.17, *p* = 0.021, η^2^ = 0.148; *post hoc* all *p* ≤ 0.046]. Latencies were increased during the combined *n*-back/nogo task in comparison to the single go/nogo task [*F*(1,24) = 40.27, *p* < 0.001, η^2^ = 0.627].

##### Nogo P3

Comparing P3 amplitudes between stimulus types of the go/nogo task, we found main effects of Stimulus [*F*(1,24) = 43.91, *p* < 0.001, η^2^ = 0.647], Region [*F*(1.5,36.3) = 91.23, *p* < 0.001, η^2^ = 0.792], and Hemisphere [*F*(1.6,37.4) = 7.92, *p* = 0.003, η^2^ = 0.248], interactions between Stimulus and Region [*F*(2,48) = 10.80, *p* < 0.001, η^2^ = 0.310], Stimulus and Hemisphere [*F*(2,48) = 21.00, *p* < 0.001, η^2^ = 0.467], Region and Hemisphere [*F*(4,96) = 6.66, *p* < 0.001, η^2^ = 0.217], and a threefold interaction [*F*(4,96) = 6.52, *p* < 0.001, η^2^ = 0.214]. Nogo stimuli elicited larger P3 components than go stimuli. This effect was greatest at central electrode positions, but was significant also at frontal and parietal sites (all *p* < 0.001). Generally, P3 amplitudes were largest at parietal and smallest at frontal positions (all *p* < 0.001). However, during nogo trials, P3 amplitudes were centro-parietally largest at midline sites (all *p* ≤ 0.026) while during go trials P3 amplitudes were frontally, largest at the right site (all *p* ≤ 0.013). The next comparison between single and combined task versions revealed interactions between Task and Region [*F*(2,48) = 9.95, *p* < 0.001, η^2^ = 0.293] as well as between Task, Region, and Hemisphere [*F*(4,96) = 3.14, *p* = 0.018, η^2^ = 0.116], indicating larger P3 amplitudes during the combined *n*-back/nogo task at midline and right parietal electrode positions (all *p* ≤ 0.007).

We found higher P3 latencies during nogo than during go trials [*F*(1,24) = 130.41, *p* < 0.001, η^2^ = 0.845]. Further, latencies were increased in the combined *n*-back/nogo task compared to the single go/nogo task [*F*(1,24) = 84.79, *p* < 0.001, η^2^ = 0.779].

### Comparisons Between Control and ADHD Group

#### Behavioral Data

As expected, group comparisons revealed that ADHD patients showed deficits in all behavioral measures during the combined *n*-back/nogo task. Working memory performance was reduced to 51.62% [controls: 73.83%, *t*(57) = 4.29, *p* < 0.001, *d* = 1.16], and response inhibition performance was reduced to 77.85% [controls: 87.44%, *t*(49.4) = 2.74, *p* = 0.009, *d* = 0.69]. Moreover, patients showed more omission errors [*t*(34.2) = −3.72, *p* = 0.001, *d* = 0.91] as well as higher reaction times [*t*(57) = −2.15, *p* = 0.036, *d* = 0.57] and higher standard deviations of reaction times [*t*(57) = −3.33, *p* = 0.002, *d* = 0.88]. Details of all comparisons are given in Table 4.

**TABLE 4:** Comparisons of behavioral data between control and ADHD group, mean ± standard deviation, *t* and *p*-values as well as effect sizes Cohens *d* are given.

	Controls	ADHD	*t*	*p*	*d*
WM in %	73.83 ± 15.39	51.62 ± 22.22	*t*(57.0) = 4.29	<0.001	1.16
RI in %	87.44 ± 8.35	77.85 ± 17.93	*t*(49.4) = 2.74	0.009	0.69
Omission errors in %	1.07 ± 1.25	7.89 ± 10.58	*t*(34.2) = −3.72	0.001	0.91
Reaction time in ms	722 ± 194	833 ± 197	*t*(57.0) = −2.15	0.036	0.57
SD of reaction time in ms	252 ± 72	316 ± 73	*t*(57.0) = −3.33	0.002	0.88

*WM, working memory (performance); RI, response inhibition (performance).*

In ADHD patients, we found a significant correlation between working memory and response inhibition performance assessed with the combined *n*-back/nogo task (*r* = 0.643, *p* < 0.001). This was not associated with reported attention problems (CBCL, YSR) or attentional performance (d2, d2-R) (all *r* ≤ 0.296, *p* ≥ 0.118).

#### ERP Data

##### n-back P3

In ADHD patients, diminished amplitudes of the *n*-back P3 component were found [*F*(1,55) = 4.44, *p* = 0.040, η^2^ = 0.075] but latency was not significantly different between the control and the ADHD group [*F*(1,55) = 0.28, *p* = 0.599, η^2^ = 0.005].

##### Nogo N2

Amplitudes of the nogo N2 component did not differ between groups [*F*(1,54) = 0.95, *p* = 0.335, η^2^ = 0.017]. However, ADHD patients showed delayed N2 latencies [*F*(1,54) = 6.34, *p* = 0.015, η^2^ = 0.105].

##### Nogo P3

A main effect of Group was identified for the nogo P3 amplitude [*F*(1,54) = 11.60, *p* = 0.001, η^2^ = 0.177] indicating a reduced peak amplitude in the ADHD group. Moreover, an interaction between Group and Hemisphere was revealed [*F*(2,108) = 3.47, *p* = 0.035, η^2^ = 0.060]. This indicated that controls showed largest P3 peaks at midline electrode positions (all *p* ≤ 0.001), while in patients differences between midline and lateral electrodes were reduced but still significant (all *p* ≤ 0.020). Further, in controls P3 peaks were larger at right compared to left electrode sites (*p* = 0.050) but this effect was missing in ADHD. The group analysis revealed no latency differences of the nogo P3 component [*F*(1,54) = 0.09, *p* = 0.761, η^2^ = 0.002].

**FIGURE 4:**
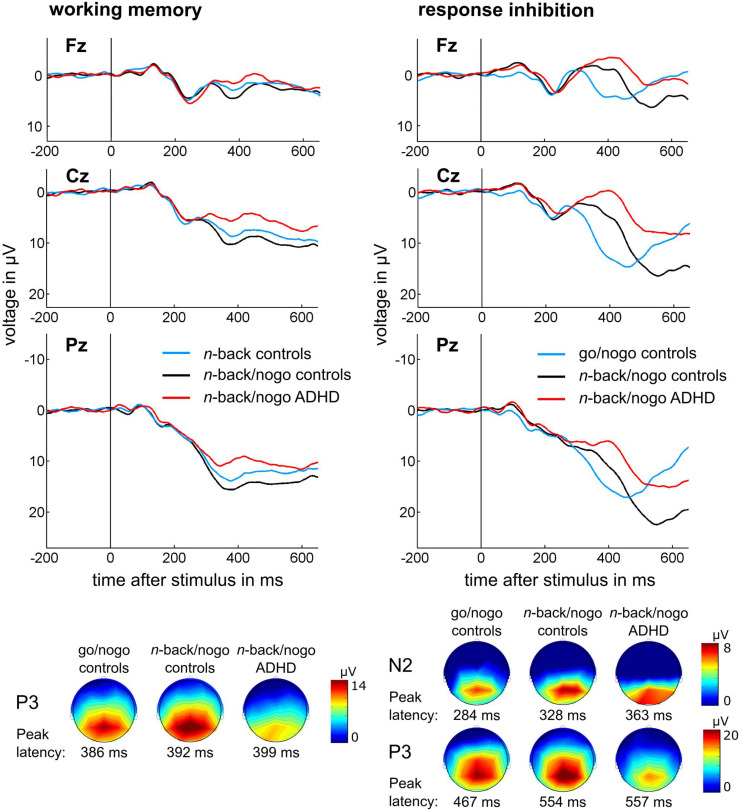
Stimulus-locked, averaged ERP waveforms and topographical plots are illustrated for (A) *n*-back target trials of the working memory task and (B) nogo trials of the response inhibition task at electrode positions Fz, Cz, and Pz, topographical plots are displayed for peak latency of the respective condition and group at Pz for *n*-back and nogo P3, and at Cz for nogo N2.

ERP data are illustrated in Figure 4 and presented in Table 5 in full detail. The results of all ANOVAs are summarized in Supplementary Table 2. The datasets of this study can be found under https://doi.org/10.6084/m9.figshare.c.4933326.

**TABLE 5:** EEG peak amplitudes and latencies for single task versions and for the combined *n*-back/nogo task in controls and ADHD patients, mean ± standard deviation.

	Amplitude in μV				Latency in ms			
	Controls			ADHD	Controls			ADHD
	Single task		*n*-back/nogo	*n*-back/nogo	Single task		*n*-back/nogo	*n*-back/nogo
*n-back P3*	*Non-target*	*Target*	*Target*	*Target*	*Non-target*	*Target*	*Target*	*Target*
C3	5.07 ± 4.05	10.16 ± 5.16	10.26 ± 6.57	7.54 ± 5.46	406 ± 64	394 ± 66	388 ± 49	385 ± 62
Cz	5.83 ± 5.38	11.61 ± 5.92	12.31 ± 6.52	8.84 ± 5.52	417 ± 70	382 ± 58	384 ± 52	375 ± 63
C4	6.17 ± 5.27	10.76 ± 6.09	11.48 ± 5.27	9.31 ± 5.19	394 ± 74	384 ± 46	383 ± 46	377 ± 66
P3	8.55 ± 4.78	14.32 ± 6.41	15.36 ± 7.17	12.05 ± 6.61	360 ± 61	399 ± 58	390 ± 53	384 ± 58
Pz	10.80 ± 5.23	16.10 ± 6.84	17.53 ± 7.33	13.79 ± 5.71	382 ± 66	386 ± 53	392 ± 51	399 ± 65
P4	10.70 ± 4.81	15.02 ± 6.18	15.59 ± 6.17	12.77 ± 5.81	371 ± 65	386 ± 50	397 ± 58	377 ± 61

***Nogo N2***	***Go***	***Nogo***	***Nogo***	***Nogo***	***Go***	***Nogo***	***Nogo***	***Nogo***

F3	–1.24 ± 3.95	–3.77 ± 5.67	–4.53 ± 4.92	–4.87 ± 6.05	284 ± 33	303 ± 35	374 ± 58	396 ± 54
Fz	–1.68 ± 4.32	–4.44 ± 5.89	–5.19 ± 5.23	–6.18 ± 6.83	288 ± 29	298 ± 35	358 ± 52	381 ± 58
F4	–0.39 ± 4.34	–2.52 ± 5.11	–4.51 ± 5.79	–5.88 ± 7.26	274 ± 33	289 ± 36	358 ± 51	383 ± 52
C3	2.59 ± 4.01	–0.40 ± 3.79	–0.81 ± 5.25	–2.40 ± 4.98	275 ± 36	296 ± 38	350 ± 57	381 ± 49
Cz	2.56 ± 6.31	–0.28 ± 4.93	–0.67 ± 5.69	–2.98 ± 5.87	284 ± 33	284 ± 33	328 ± 55	363 ± 55
C4	2.73 ± 4.73	–0.72 ± 4.56	–1.11 ± 5.76	–2.95 ± 5.91	269 ± 40	288 ± 37	334 ± 57	368 ± 58

***Nogo P3***	***Go***	***Nogo***	***Nogo***	***Nogo***	***Go***	***Nogo***	***Nogo***	***Nogo***

F3	2.26 ± 4.42	7.29 ± 6.09	7.72 ± 5.70	4.42 ± 5.42	317 ± 63	438 ± 68	539 ± 52	541 ± 56
Fz	2.07 ± 4.28	8.57 ± 7.49	9.13 ± 6.70	4.19 ± 6.02	328 ± 62	443 ± 57	535 ± 49	542 ± 39
F4	4.07 ± 4.35	8.34 ± 6.81	7.99 ± 6.18	3.93 ± 6.48	317 ± 58	443 ± 61	531 ± 49	537 ± 53
C3	6.48 ± 4.70	13.20 ± 8.14	14.40 ± 7.51	8.57 ± 5.73	329 ± 54	450 ± 61	556 ± 51	557 ± 46
Cz	6.66 ± 5.44	17.76 ± 10.11	18.81 ± 8.82	10.94 ± 7.23	315 ± 61	467 ± 57	551 ± 49	550 ± 49
C4	7.38 ± 5.49	15.13 ± 8.79	16.20 ± 9.01	9.66 ± 6.33	324 ± 54	474 ± 58	553 ± 46	553 ± 46
P3	11.23 ± 5.44	15.72 ± 7.64	17.72 ± 7.40	14.00 ± 6.61	322 ± 44	461 ± 53	560 ± 46	566 ± 48
Pz	11.86 ± 6.00	19.70 ± 8.68	24.61 ± 9.45	17.70 ± 7.60	327 ± 45	467 ± 55	554 ± 46	557 ± 47
P4	11.14 ± 5.16	17.53 ± 7.58	21.42 ± 8.83	15.51 ± 7.46	316 ± 37	461 ± 55	546 ± 53	548 ± 52

## Discussion

In the present study, we introduced the *n*-back/nogo paradigm that combines working memory and response inhibition aspects and hypothesized that its behavioral and ERP characteristics would be comparable to those of parallel single task versions. Contrary to our expectations, working memory performance was higher in the combined *n*-back/nogo task than in the single *n*-back task but still, both measures were highly correlated. We confirmed that response inhibition performance was similar between the combined *n*-back/nogo and the single go/nogo task version. Further, we found that reaction times and standard deviations of reaction times in the combined *n*-back/nogo task were comparable to those of the single *n*-back task but were higher than those of the single go/nogo task. As expected, the combined *n*-back/nogo paradigm demonstrated comparable ERP structures as single task versions for working memory and response inhibition task aspects. Still, we found larger nogo P3 amplitudes as well as increased nogo N2 and nogo P3 latencies for the combined task. The application of the combined paradigm in ADHD patients revealed the expected working memory and response inhibition deficits, increased omission errors, reaction times, and standard deviations of reaction time, as well as diminished *n*-back P3 and nogo P3 amplitudes. However, we found no reduction of the nogo N2 amplitude in patients.

In healthy individuals, working memory performance was found to be better during the combined *n*-back/nogo task compared to the single *n*-back task. Despite this difference in performance outcome, we argue that the combined task delivers a valid measurement of working memory, because working memory performance was highly correlated between combined and single task versions. Therefore, the improved performance outcome seems to result from a reduced difficulty of the combined paradigm. This is remarkable, as previous studies found that working memory performance decreased when inhibitory task aspects were added (Alderson et al., 2017). The performance decline that was found in prior studies resulted probably from the fact that participants were required to keep more rules in mind in order to meet task demands. However, in the present study the combination of paradigms caused a decrease in task difficulty of the working memory aspect, which we assume resulted from the introduction of nogo trials. The nogo stimuli itself were not part of the *n*-back sequence. Further, after each nogo trial a new sequence of *n*-back letters started and hence, the first two letters only needed to be encoded but not matched to previous letters (Chen et al., 2008). Thus, working memory load was reduced in this task, which could have caused the improved task performance. Generally, the 2-back paradigm is a challenging task, even for healthy individuals, indicated by a performance rate of only 56% in our study for the single task version. In ADHD patients, worse working memory performance would be expected, as in Alderson et al. (2017) who reported values as low as 27% in children with ADHD. Such low performance could cause a dramatic drop in motivation and therefore, compromise compliance if participants perceive the task as too difficult. Furthermore, we showed reduced omission errors during the combined *n*-back/nogo task compared to the single *n*-back task version, further suggesting that the difficulty during the combined task version was appropriate to induce high levels of sustained attention together with stable task performance (Thomson et al., 2015). A high number of accurate trials is needed for data analysis in neurophysiological and imaging studies. Accordingly, reduced difficulty of the combined *n*-back/nogo task should be advantageous for the investigation of populations with working memory impairments, as ADHD patients. Moreover, applications in investigations of autism spectrum disorder or young children are conceivable.

In accordance with our hypothesis, we found no difference in response inhibition performance between the single go/nogo and the combined *n*-back/nogo task. Moreover, response inhibition assessment was correlated between single and combined task versions, albeit only after the exclusion of two outliers. Although these results require a careful interpretation, we conclude that the combined *n*-back/nogo task is suitable to investigate response inhibition. Still, the analysis suggests that significant portions of variance between task versions remain unexplained. We speculate that increased reaction times in the combined *n*-back/nogo task compared to the single go/nogo task version accounted for parts of this variance. It is conceivable that slowed responses were associated with changes in stimulus processing or task strategies. For example, such effects were demonstrated in the context of a negative priming task (Mayr et al., 2006). However, it remains to be clarified how the dual-task requirements of the combined paradigm accounted for variability between task versions.

The investigation of discriminant validity in healthy participants demonstrated no correlation between measures of working memory and response inhibition within the combined *n*-back/nogo task. This matches the findings of single *n*-back and go/nogo tasks, which showed no correlation between working memory and response inhibition as well, although only after the exclusion of outliers. Thus, we assume that the combined *n*-back/nogo task was suitable to measure distinct cognitive functions instead of a general task factor.

While response inhibition performance was mostly constant between task versions, we demonstrated slowed reaction times during the combined task compared to the single go/nogo task. This was reflected by significant differences in the IES. We think the reason is that reactions during the single go/nogo task required only one-digit button presses whereas all reactions during the combined *n*-back/nogo task demanded working memory decisions. In addition, complex task demands in the combined *n*-back/nogo task have probably resulted in less automated prepotent response tendencies, leading to slower reaction times. For these reasons, it must be considered that reaction times of the combined *n*-back/nogo task are rather equivalent to such of an *n*-back than of a go/nogo task. This conclusion is further supported by the exploratory investigation of reaction time distributions that depict more similarity to the single *n*-back than to the single go/nogo task. Still, reaction time measures of the combined *n*-back/nogo task were correlated not only to those of the single *n*-back task as but also to those of the single go/nogo task. Thus, although the absolute values differed between task versions, the relation between participants was preserved. This implies that reaction time comparisons, for example between groups of individuals or conditions, should result in the same conclusions, whether conducted with the combined *n*-back/nogo task or with a single go/nogo task, only differing in absolute values. However, this assumption needs to be validated in further investigations.

The combined *n*-back/nogo task evoked an *n*-back P3 component with similar amplitude and latency as the single *n*-back task, indicating analog working memory processing between task versions. In particular, comparability of amplitude size suggests that allocation of cognitive resources to working memory processes was similar in both tasks and therefore cognitive effort was similar between task versions (Dong et al., 2015). We stated earlier that difficulty of the working memory task aspect was lower for the combined than for the single task version. We assume that during the easier combined task version working memory processing was already at its full capacity. Hence, higher task difficulty during the single *n*-back task resulted in a ceiling effect, because no increase of cognitive engagement was possible in order to meet task demands. Instead, the performance dropped. Once again, this suggests that difficulty of the combined *n*-back/nogo task was adequate for our sample as it produced maximum cognitive engagement at a high level of performance.

The nogo N2 component that was elicited during the combined *n*-back/nogo task, had a similar peak amplitude as during the single go/nogo task, indicating similar effectiveness of conflict monitoring (Donkers and van Boxtel, 2004; Jonkman, 2006; Smith et al., 2008). However, the nogo P3 component evoked during the combined *n*-back/nogo task had a larger peak amplitude compared to the single task version at midline and right parietal electrode positions. Topographic plots demonstrated that the nogo P3 showed a parietal distribution that was more pronounced in the right hemisphere, which corresponds to the area where differences between task versions were detected. Larger peaks of the nogo P3 have been associated with better response inhibition (Donkers and van Boxtel, 2004; Jonkman, 2006; Smith et al., 2008). Accordingly, we assume that more resources were allocated to this process during the combined *n*-back/nogo task. We hypothesize that differences in amplitude size were associated with increased reaction times in the combined *n*-back/nogo task, because it was demonstrated that ERP responses can become more pronounced with prolonged reaction times (Mayr et al., 2006). Thus, we assume that the slowed responding allowed for the allocation of more resources to the process of response inhibition.

Increased latencies of nogo N2 and P3 components were found during the combined *n*-back/nogo task. Component latencies indicate the speed of stimulus evaluation and thus, increase with growing task-processing demands (Polich, 2007; Gajewski and Falkenstein, 2013). We therefore, assume that increased latencies of nogo related components reflect higher demands for stimulus processing linked to less automated responding during the combined task version caused by the working memory aspect. As component latencies are associated with response time (Polich, 2007), this ERP result matches behavioral findings of higher reaction times during the combined *n*-back/nogo task.

ADHD patients demonstrated impaired performance in all behavioral measures using the combined *n*-back/nogo paradigm. We showed impaired working memory and response inhibition performance in patients, as it was expected from prior research with *n*-back (Kasper et al., 2012; Myatchin et al., 2012) and go/nogo tasks (Wodka et al., 2007; Neely et al., 2017). In contrast to the healthy control group, there was a high correlation between working memory and response inhibition performance in patients, which implies that both cognitive functions were not assessed independently with the combined *n*-back/nogo task. This could not be explained with a mediation by ADHD symptom severity as we found no association with diagnostic information. However, a relation between executive deficits in working memory and response inhibition has been demonstrated in ADHD (Clark et al., 2007; Schecklmann et al., 2013), and seems to be associated specifically to an underactivation of right frontal brain regions (Clark et al., 2007). Thus, we hypothesize that the correlation between working memory and response inhibition impairments demonstrated in the present study, could reflect a general underlying deficit in executive functioning in ADHD. Still, as ADHD patients did not solve single task versions, this topic could not be explored further and our conclusions are consequently limited. As it was expected, ADHD patients showed increased numbers of omission errors, which has been associated with inattentive and hyperactive/impulsive symptom severity (Epstein et al., 2003; Bezdjian et al., 2009). Moreover, ADHD related increases in reaction times and in standard deviations of reaction time were found using the combined *n*-back/nogo task (Salum et al., 2019). We thus conclude that the introduced *n*-back/nogo task was suitable to assess executive deficits in ADHD.

Neurophysiological results from ADHD patients further reinforced this conclusion. Using the combined *n*-back/nogo task, we found diminished *n*-back P3 amplitudes in patients, as it was expected from prior research. This finding reflects ADHD related working memory deficits on a neurophysiological level (Barry et al., 2003; Keage et al., 2008; Szuromi et al., 2011; Johnstone et al., 2013). Unexpectedly, amplitudes of the nogo N2 component did not differ between patients and controls. However, previous research showed heterogeneous results for this component (Fallgatter et al., 2004; Shahaf et al., 2015). In addition, a recent meta-analysis by Kaiser et al. (2020) could not confirm amplitude reductions for this component and concluded that reduced amplitudes of the nogo N2 could be characteristic for a subgroup of the ADHD population. Indeed, pronounced N2 reductions were found particularly in younger ADHD patients of about 10 years (Johnstone et al., 2013). Thus, it is possible that we missed this effect because our sample was older. Therefore, it remains to be clarified, if the subpopulations of patients with reduced nogo N2 components would show this reduction also with the combined *n*-back/nogo task. Again, the nogo P3 component showed the expected amplitude reduction in patients reflecting ADHD related response inhibition deficits (Smith et al., 2008; Fisher et al., 2011). Regarding latency alterations of the investigated components, prior research has been inconclusive in ADHD (Fallgatter et al., 2004; Smith et al., 2008; Johnstone et al., 2009; Fisher et al., 2011). We found increased latencies of the nogo N2 in patients, which could be related to the behavioral finding of slowed reaction times (Gajewski and Falkenstein, 2013).

Two characteristics of the ADHD sample investigated in the present study are of importance for the interpretation of results, namely age and comorbidities. ADHD patients in this study had a mean age of 13 years. As cognitive impairments in ADHD and neurophysiologic correlates vary with age (Marx et al., 2010; Kaiser et al., 2020) it is possible that the sensitivity of the introduced paradigm for cognitive deficits depends on the investigated age group. Further, in this study patients with comorbid disorders were excluded, because they differ from patients with ADHD only, in cognitive and ERP profiles (Banaschewski et al., 2003; Noordermeer et al., 2015). Although this study design was favorable to reduce variability in the ADHD sample, comorbid disorders as oppositional defiant disorder and conduct disorder occur in up to half of ADHD patients (DSM-5; American Psychiatric Association, 2013). Thus, future experiments remain to demonstrate applicability of the introduced *n*-back/nogo task in ADHD samples with different characteristics.

As a limitation, single task versions of *n*-back and go/nogo paradigms were applied only in healthy participants, which allows no direct conclusions on differences between task versions in ADHD and which should be addressed in future research. Further, ADHD data were pooled from the context of two different studies. However, we expect that this procedure increased variability in the data, hampering to demonstrate the expected effects. As the results were still in line with our hypotheses, this indicates that the effects are robust under different experimental conditions. A strength of this experiment was the investigation of ADHD patients without comorbidities because this excludes confounded effects from other disorders than ADHD (Banaschewski et al., 2003).

## Conclusion

In the present ERP study, we introduced the combined *n*-back/nogo paradigm and demonstrated its effectiveness for the assessment of working memory and response inhibition deficits in children and adolescents with ADHD on a behavioral and neurophysiological level. As both executive functions can be assessed during the same task, this paradigm provides an economical alternative to single task versions. Thus, we emphasize its relevance for research in ADHD patients and other populations who require short experimental procedures, for example in the context of developmental research with younger children and elderly people, or in clinical populations with developmental disorders. Conceivable are further applications in settings where working memory and response inhibition brain areas should be activated simultaneously, for instance in cognitive trainings or during non-invasive brain stimulation.

## Data Availability Statement

The datasets presented in this study can be found in online repositories (https://doi.org/10.6084/m9.figshare.c.4933326). The names of the repository/repositories and accession number(s) can be found in the article/Supplementary Material.

## Ethics Statement

The studies involving human participants were reviewed and approved by the Ethics Committee, Otto von Guericke University Magdeburg. Written informed consent to participate in this study was provided by the participants’ legal guardian/next of kin.

## Author Contributions

CB-Z and KK conceived of the idea in the present study. KK and JT established the diagnostic procedure of participants. CB-Z and JT developed the paradigms. CB-Z implemented the tasks, carried out most of the experiments, and performed the data analysis. CB-Z and KK shaped the analysis. All the authors contributed to the interpretation of the data. CB-Z prepared the manuscript. JT, H-HF, and KK contributed to the final version of the manuscript.

## Conflict of Interest

The authors declare that the research was conducted in the absence of any commercial or financial relationships that could be construed as a potential conflict of interest.

## Footnotes

1 www.neurobs.com
