# Supplementary material for: Economical Assessment of Working Memory and Response Inhibition in ADHD Using a Combined n-back/Nogo Paradigm: An ERP Study
Source: Front Hum Neurosci. 2020 Aug 6;14:322. doi: 10.3389/fnhum.2020.00322 (PMC7426064; doi:10.3389/fnhum.2020.00322)
Supplement: Supplementary file 1 [file Data_Sheet_1.PDF]

Supplementary Material

Supplementary Table 1. Comparisons between control and ADHD groups, given separately for two different studies from which ADHD data were pooled, mean ± standard deviation.

		ADHD (n = 24)			ADHD (n = 10)		
	controls	values	*t*	*p*	values	*t*	*p*
WM in %	73.83 ± 15.39	49.43 ± 22.76	*t*(47.0) = 4.41	< .001	56.87 ± 21.07	*t*(33.0) = 2.65	.012
RI in %	87.44 ± 8.35	73.52 ± 19.42	*t*(31.0) = 3.24	.003	88.25 ± 6.76	*t*(33.0) = -0.27	.788
omission errors in %	1.07 ± 1.25	10.14 ± 11.89	*t*(23.5) = -3.72	.001	2.47 ± 1.81	*t*(12.6) = -2.25	.043
reaction time in ms	722 ± 194	839 ± 206	*t*(47.0) = -2.05	.046	819 ± 182	*t*(33.0) = -1.35	.186
SD of reaction time in ms	252 ± 72	330 ± 69	*t*(47.0) = -3.86	< .001	282 ± 74	*t*(33.0) = -1.09	.284
n-back P3 amplitude at Pz in µV	17.53 ± 7.33	14.56 ± 4.77	*t*(47.0) = 1.67	.101	11.49 ± 7.83	*t*(31.0) = 2.00	.054
nogo N2 amplitude at Cz in µV	-0.67 ± 5.69	-2.76 ± 5.68	*t*(45.0) = 1.26	.215	-3.50 ± 6.66	*t*(32.0) = 1.23	.229
nogo P3 amplitude at Cz in µV	18.81 ± 8.82	12.59 ± 7.12	*t*(45.0) = 2.64	.012	6.88 ± 6.09	*t*(32.0) = 3.73	.001
n-back P3 latency at Pz in ms	392 ± 51	395 ± 69	*t*(42.4) = -0.16	.878	409 ± 56	*t*(31.0) = -0.79	.437
nogo N2 latency at Cz in ms	328 ± 55	365 ± 54	*t*(45.0) = -2.36	.023	356 ± 59	*t*(32.0) = -1.30	.204
nogo P3 latency at Cz in ms	551 ± 49	557 ± 42	*t*(45.0) = -0.47	.640	532 ± 62	*t*(32.0) = 0.91	.368

WM – working memory performance, RI – response inhibition performance

Supplementary Table 2. Results of ANOVAs for ERP analysis, *F* and *p*-values as well as effect sizes η² are given.

Condition / Comparison	Stimulus<br>go vs. nogo / target vs. non-target	Task<br>single vs. combined	Group<br>control vs. ADHD
n-back P3 amplitude			
condition	$F(1, 24) = 45.54$, $p < .001$, $\eta^2 = .655$	$F(1, 24) = 0.83$, $p = .372$, $\eta^2 = .033$	$F(1, 55) = 4.44$, $p = .040$, $\eta^2 = .075$
region	$F(1, 24) = 79.25$, $p < .001$, $\eta^2 = .768$	-	-
hemisphere	$F(1.4, 34.0) = 6.72$, $p = .008$, $\eta^2 = .219$	-	-
condition x region	$F(1, 24) < 0.01$, $p = .966$, $\eta^2 < .001$	$F(1, 24) = 1.35$, $p = .256$, $\eta^2 = .053$	$F(1, 55) = 0.24$, $p = .624$, $\eta^2 = .004$
condition x hemisphere	$F(2, 48) = 3.33$, $p = .044$, $\eta^2 = .122$	$F(2, 48) = 0.44$, $p = .644$, $\eta^2 = .018$	$F(2, 110) = 0.77$, $p = .465$, $\eta^2 = .014$
region x hemisphere	$F(2, 48) = 1.346$, $p = .270$, $\eta^2 = .053$	-	-
condition x region x hemisphere	$F(2, 48) = 1.44$, $p = .0246$, $\eta^2 = .057$	$F(1.5, 36.9) = 1.45$, $p = .246$, $\eta^2 = .057$	$F(2, 110) = 0.14$, $p = .866$, $\eta^2 = .003$
n-back P3 latency			
condition	$F(1, 24) < 0.01$, $p = .977$, $\eta^2 < .001$	$F(1, 24) < 0.01$, $p = .964$, $\eta^2 < .001$	$F(1, 55) = 0.28$, $p = .599$, $\eta^2 = .005$
region	$F(1, 24) = 3.54$, $p = .072$, $\eta^2 = .129$	-	-
hemisphere	$F(2, 48) = 1.02$, $p = .369$, $\eta^2 = .041$	-	-
condition x region	$F(1, 24) = 10.35$, $p = .004$, $\eta^2 = .301$	$F(1, 24) = 0.21$, $p = .654$, $\eta^2 = .009$	$F(1, 55) < 0.01$, $p = .996$, $\eta^2 < .001$
condition x hemisphere	$F(2, 48) = 5.41$, $p = .008$, $\eta^2 = .184$	$F(1.6, 38.9) = 0.92$, $p = .388$, $\eta^2 = .037$	$F(2, 110) = 0.58$, $p = .561$, $\eta^2 = .010$
region x hemisphere	$F(2, 48) = 0.31$, $p = .733$, $\eta^2 = .013$	-	-

condition x region x hemisphere	$F(2, 48) = 0.61$, $p = .548$, $\eta^2 = .025$	$F(1.5, 36.4) = 0.40$, $p = .618$, $\eta^2 = .016$	$F(2, 110) = 1.63$, $p = .200$, $\eta^2 = .029$
nogo N2 amplitude			
condition	$F(1, 24) = 7.59$, $p = .011$, $\eta^2 = .240$	$F(1, 24) = 0.52$, $p = .479$, $\eta^2 = .021$	$F(1, 54) = 0.95$, $p = .335$, $\eta^2 = .017$
region	$F(1, 24) = 33.33$, $p < .001$, $\eta^2 = .581$	-	-
hemisphere	$F(2, 48) = 1.74$, $p = .186$, $\eta^2 = .068$	-	-
condition x region	$F(1, 24) = 1.03$, $p = .321$, $\eta^2 = .041$	$F(1, 24) = 1.79$, $p = .194$, $\eta^2 = .069$	$F(1, 54) = 1.21$, $p = .276$, $\eta^2 = .022$
condition x hemisphere	$F(1.6, 37.6) < 0.01$, $p = .996$, $\eta^2 < .001$	$F(2, 48) = 0.74$, $p = .481$, $\eta^2 = .030$	$F(2, 108) = 0.70$, $p = .497$, $\eta^2 = .013$
region x hemisphere	$F(2, 48) = 7.02$, $p = .002$, $\eta^2 = .226$	-	-
condition x region x hemisphere	$F(1.5, 36.8) = 0.94$, $p = .376$, $\eta^2 = .038$	$F(2, 48) = 3.05$, $p = .057$, $\eta^2 = .113$	$F(2, 108) = 0.67$, $p = .513$, $\eta^2 = .012$
nogo N2 latency			
condition	$F(1, 24) = 6.13$, $p = .021$, $\eta^2 = .204$	$F(1, 24) = 40.27$, $p < .001$, $\eta^2 = .627$	$F(1, 54) = 6.34$, $p = .015$, $\eta^2 = .105$
region	$F(1, 24) = 4.71$, $p = .040$, $\eta^2 = .164$	-	-
hemisphere	$F(2, 48) = 4.17$, $p = .021$, $\eta^2 = .148$	-	-
condition x region	$F(1, 24) = 0.03$, $p = .858$, $\eta^2 = .001$	$F(1, 24) = 2.59$, $p = .121$, $\eta^2 = .097$	$F(1, 54) = 0.84$, $p = .365$, $\eta^2 = .015$
condition x hemisphere	$F(2, 48) = 2.94$, $p = .062$, $\eta^2 = .109$	$F(2, 48) = 0.60$, $p = .552$, $\eta^2 = .024$	$F(2, 108) = 0.07$, $p = .936$, $\eta^2 = .001$
region x hemisphere	$F(2, 48) = 0.83$, $p = .442$, $\eta^2 = .033$	-	-
condition x region x hemisphere	$F(1.5, 36.4) = 0.73$, $p = .452$, $\eta^2 = .030$	$F(2, 48) = 0.18$, $p = .834$, $\eta^2 = .008$	$F(2, 108) < 0.01$, $p = .991$, $\eta^2 < .001$

nogo P3 amplitude			
condition	$F(1, 24) = 43.91$, $p < .001$, $\eta^2 = .647$	$F(1, 24) = 1.33$, $p = .260$, $\eta^2 = .053$	$F(1, 54) = 11.60$, $p = .001$, $\eta^2 = .177$
region	$F(1.5, 36.3) = 91.23$, $p < .001$, $\eta^2 = .792$	-	-
hemisphere	$F(1.6, 37.4) = 7.92$, $p = .003$, $\eta^2 = .248$	-	-
condition x region	$F(2,48) = 10.80$, $p < .001$, $\eta^2 = .310$	$F(2, 48) = 9.95$, $p < .001$, $\eta^2 = .293$	$F(2, 108) = 1.53$, $p = .222$, $\eta^2 = .027$
condition x hemisphere	$F(2, 48) = 21.00$, $p < .001$, $\eta^2 = .467$	$F(2, 48) = 1.04$, $p = .360$, $\eta^2 = .042$	$F(2, 108) = 3.47$, $p = .035$, $\eta^2 = .060$
region x hemisphere	$F(4, 96) = 6.66$, $p < .001$, $\eta^2 = .217$	-	-
condition x region x hemisphere	$F(4, 96) = 6.52$, $p < .001$, $\eta^2 = .214$	$F(4, 96) = 3.14$, $p = .018$, $\eta^2 = .116$	$F(4, 216) = 0.83$, $p = .505$, $\eta^2 = .015$
nogo P3 latency			
condition	$F(1, 24) = 130.41$, $p < .001$, $\eta^2 = .845$	$F(1, 24) = 84.79$, $p < .001$, $\eta^2 = .779$	$F(1, 54) = 0.09$, $p = .761$, $\eta^2 = .002$
region	$F(1.6,38.6) = 1.69$, $p = .201$, $\eta^2 = .066$	-	-
hemisphere	$F(2, 48) = 0.67$, $p = .518$, $\eta^2 = .027$	-	-
condition x region	$F(1.5, 36.3) = 1.61$, $p = .215$, $\eta^2 = .063$	$F(1.2, 28.3) = 0.05$, $p = .861$, $\eta^2 = .002$	$F(2, 108) = 0.09$, $p = .916$, $\eta^2 = .002$
condition x hemisphere	$F(2, 48) = 1.72$, $p = .190$, $\eta^2 = .067$	$F(1.5, 36.1) = 3.45$, $p = .055$, $\eta^2 = .126$	$F(2, 108) < 0.01$, $p = .999$, $\eta^2 < .001$
region x hemisphere	$F(4, 96) = 1.54$, $p = .197$, $\eta^2 = .060$	-	-
condition x region x hemisphere	$F(4, 96) = 1.41$, $p = .236$, $\eta^2 = .055$	$F(4, 96) = 0.38$, $p = .822$, $\eta^2 = .016$	$F(4, 216) = 0.17$, $p = .953$, $\eta^2 = .003$
